# Erythropoietin in Brain Development and Beyond

**DOI:** 10.1155/2012/953264

**Published:** 2012-02-26

**Authors:** Mawadda Alnaeeli, Li Wang, Barbora Piknova, Heather Rogers, Xiaoxia Li, Constance Tom Noguchi

**Affiliations:** Molecular Medicine Branch, National Institute of Diabetes and Digestive and Kidney Diseases, National Institutes of Health, Bethesda, MD 20892-1822, USA

## Abstract

Erythropoietin is known as the requisite cytokine for red blood cell production. Its receptor, expressed at a high level on erythroid progenitor/precursor cells, is also found on endothelial, neural, and other cell types. Erythropoietin and erythropoietin receptor expression in the developing and adult brain suggest their possible involvement in neurodevelopment and neuroprotection. During ischemic stress, erythropoietin, which is hypoxia inducible, can contribute to brain homeostasis by increasing red blood cell production to increase the blood oxygen carrying capacity, stimulate nitric oxide production to modulate blood flow and contribute to the neurovascular response, or act directly on neural cells to provide neuroprotection as demonstrated in culture and animal models. Clinical studies of erythropoietin treatment in stroke and other diseases provide insight on safety and potential adverse effects and underscore the potential pleiotropic activity of erythropoietin. Herein, we summarize the roles of EPO and its receptor in the developing and adult brain during health and disease, providing first a brief overview of the well-established EPO biology and signaling, its hypoxic regulation, and role in erythropoiesis.

## 1. Introduction

Erythropoietin (EPO) is produced primarily in the adult kidney and secreted into the circulation to regulate red blood cell production in the bone marrow. EPO stimulates erythroid progenitor cell survival, proliferation, and differentiation to satisfy the daily requirement of about 200 billion new red blood cells due in part to the limited red blood cell lifespan of 120 days. Human recombinant EPO has been used clinically for more than 2 decades to treat anemia associated with conditions such as chronic kidney disease, antiviral HIV therapy, and cancer patients on chemotherapy. EPO production is hypoxia inducible and thus increases during anemia and hypoxic stress. Interestingly, EPO production has also been detected in brain in response to hypoxic stress. The finding that EPO receptor (EpoR) expression extends beyond hematopoietic tissue to include neural and endothelial cells and the accumulating evidence for EPO antiapoptotic properties such as its neuroprotective activity have collectively led to investigations of EPO as a pleiotropic cytokine. In this paper, we review the nonhematopoietic activity of EPO in the developing as well as adult brain, and summarize its roles during health and disease.

## 2. EPO, EpoR Signaling, and Erythropoiesis

**Figure 1:**
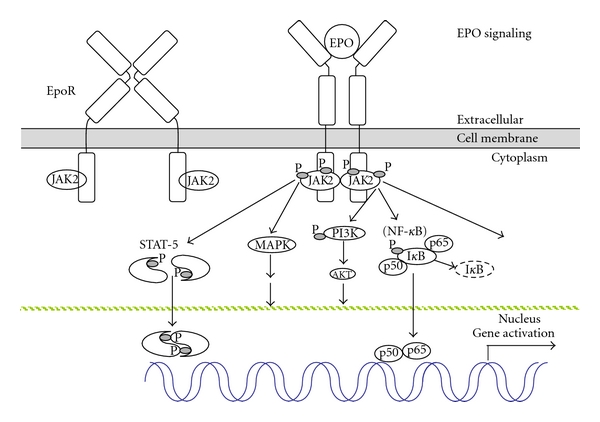
Erythropoietin signaling. EPO binding to the homodimeric EpoR on the cell surface changes the conformation of EpoR and brings the respective cytoplasmic domains in closer proximity resulting in transphosphorylation and activation of the associated Janus kinase JAK2 proteins. JAK2 activation results in phosphorylation, dimerization, and translocation of signal transducer and activator of transcription (STAT) proteins, and activation of other downstream signaling pathways such as mitogen-activated protein kinase (MAPK), phosphoinositide 3-kinase (PI3K/AKT), and, in neuronal cells, nuclear factor-(NF-) *κ*B (p50 and p65) with dissociation and degradation of the inhibitory I*κ*B protein.

EPO is a glycoprotein hormone consisting of a single polypeptide of 166 amino acids folded into four *α*-helices with two disulphide bridges between cysteines 6 and 161 and between cysteines 29 and 33 [1–3]. EPO is composed of 40% to 60% carbohydrate, with a molecular mass of 30 to 34 kDa, depending on carbohydrate content. Three *N*-glycosylation sites at asparagines 24, 38, and 83 can each accommodate up to four sialic residues and one *O*-glycosylation site at serine 126 (absent in rodent EPO), which does not appear to be necessary for EPO activity [4]. Nonsialated EPO is rapidly cleared from the circulation via the galactose receptor in the liver [5]. EPO shares structural similarities with growth hormone and other members of the hematopoietic class 1 cytokine superfamily that include several interleukins (e,g, IL-2, -3, -4, -6), granulocyte-colony-stimulating factor, thrombopoietin, prolactin, oncostatin M, ciliary neurotrophic factor, and leukocyte inhibitory factor [6, 7]. The corresponding receptors for the hematopoietic class 1 cytokines are single transmembrane polypeptides that associate as homodimers, as is the case with EpoR, heterodimers, or heterotrimers. These receptors have no intrinsic catalytic domains and their cytoplasmic regions associate with Janus kinases (JAK) such as JAK2 for EPO signaling [8]. The extracellular domain of EpoR has a short *α*-helix preceding two seven-stranded *β*-sheet domains, five cysteine residues, and a Trp-Ser-X-Trp-Ser motif proximal to the membrane and characteristic of receptors for the hematopoietic class 1 cytokines [9]. The human *EpoR* gene product is encoded from 8 exons spanning over 6.5 kb, creating a 508 amino acid protein [10]. The single transmembrane domain is encoded in exon 6 with much of the cytoplasmic domain encoded in exon 8 [9, 11]. JAK2 binds to EpoR at its Box1/Box2 regions, which allows JAK2 to move from an inactive state when not in contact with EpoR to an active state following EPO stimulation [12]. EPO binding to EpoR on the surface of early erythroid progenitor cells causes a conformational change in the cytoplasmic domain bringing the two associated JAK2 proteins in close proximity leading to transphosphorylation of JAK2 and EpoR and activation of downstream signal transduction pathways (Figure 1) [13]. Notably, the extent of cell surface EpoR expression determines EPO response.

Targeted deletion in mice of either EPO or EpoR leads to a marked decrease in circulating primitive erythroblasts in utero by day E11.5, and definitive erythroid progenitor cells at the CFU-E (colony forming unit-erythroid) stage do not survive resulting in severe anemia and death at day E13.5 [14, 15]. Studies in rodents have been useful in understanding erythropoiesis during mammalian development, where differentiation of hematopoietic stem cells to form erythrocytes is initiated extra embryonically. In mice, primitive erythropoiesis begins in the extra embryonic yolk sac at around embryonic day E7.5 where EpoR transcripts are also detected [16]. Primitive erythroid cells are large, nucleated, and express embryonic globins. In the embryo proper, hematopoietic stem cells initiate in the aorta-gonad-mesonephros region around day E10 and subsequently colonizes the fetal liver [17]. By day E12.5, definitive erythropoiesis is well established in the fetal liver. Definitive erythrocytes are enucleated, are smaller than the nucleated primitive erythroid cells, and express fetal (human) or adult (mouse) globins [18]. EPO acts as a survival factor for both primitive and definitive erythroid progenitor cells and stimulates erythropoiesis by binding to its cell surface receptor [19]. EPO is actively produced in the fetal liver, decreases with embryonic maturation, and is minimal and difficult to detect by day E17.5 in mice, concomitant with the rapid decrease of erythropoiesis in the fetal liver and onset of erythropoiesis in fetal spleen accompanied by EPO expression in fetal spleen and kidney [20]. After birth, the bone marrow and, in mice, the spleen become the primary sites of erythropoiesis [21].

## 3. Sites of EPO Production

EPO is produced in the fetal liver in hepatocytes surrounding central veins and in fibroblast-like Ito cells [22]. Meanwhile, EPO production in the kidney begins before birth, increasing sharply after 30 weeks in human and localizes to the peritubular interstitial cells with neural characteristics [21, 23, 24]. Although primary EPO production switches to the adult kidney after birth, hepatic EPO mRNA can increase to 20%–50% of total body EPO mRNA in response to hypoxic-challenge [25, 26]. Hypoxia inducible factor (HIF) is the primary regulatory factor for hypoxic induction of EPO resulting in increased production of 150-fold or more. An HIF motif that regulates induction by hypoxia in the liver is located immediately 3′ of the *EPO* gene, and regulatory elements necessary for hypoxic induction in the kidney are located 6–14 kb 5′ [22, 27]. In the kidney, hypoxic induction results in increased number of cells expressing EPO, while hypoxic induction in the liver increases the amount of EPO expression per hepatocyte [23, 28].

Beside the kidney and fetal liver, EPO production is also detected in the reproductive tract and the central nervous system (CNS). In female rodents, EPO is produced in the endometrium in a hypoxia-inducible and estrogen-dependent manner [29, 30]. In male rodents, major sites of EPO mRNA production in the testis are the Sertoli and peritubular myoid cells [31]. EPO mRNA is expressed in the epididymis, is hypoxia inducible, and increases dramatically with age and sexual maturation [32].

Interestingly, EPO levels in the CNS do not follow EPO levels in the circulation. Astrocytes produce EPO, pointing to the possibility that EPO can be available on both sides of the blood-brain barrier [33, 34]. Not surprisingly, EPO production in brain is also hypoxia inducible and can persist for up to 24 hr or more [30]. EPO expression in the CNS is observed as early as 5 weeks postconception and increases with development [35–38]. By 7 weeks, EPO is detected in the spinal cord and localizes midtrimester to ependymal cells. Hypoxia-inducible EPO is also expressed in the retina [39]; during the first two trimesters of human development, EPO is detected in the retina and adrenal cortex [40]. EPO production localizing to astrocytes and neurons persists through development and adulthood, decreasing with age [36, 41]. Moreover, hypoxia-induced EPO production in astrocytes, in adult human, nonhuman primate, and rodents has been localized in various brain areas including the hippocampus, amygdale, and temporal cortex [33].

## 4. Hypoxic Regulation of EPO

EPO production is regulated primarily at the transcription level. The HIF binding site located within the highly conserved 3′ hypoxia-responsive element (HRE) of the *EPO* gene is necessary for hypoxia induction in hepatocytes [27, 42–44]. HIF is an important regulatory factor for the activation of genes in response to physiologic stress or hypoxia. The HRE also provides for hypoxic regulation by transcription factors including hepatocyte nuclear factor 4, an orphan nuclear receptor expressed in kidney and liver [45]. HIF is a heterodimer that consists of a constitutive *β*-subunit (also called aryl hydrocarbon receptor nuclear translocator protein; ARNT) that resides in the nucleus and an *α*-subunit (HIF-1*α*, HIF-2*α*, and HIF-3*α*) localized to the cytoplasm, which is transported to the nucleus for HIF activation [46, 47]. Hypoxia induction of EPO was initially thought to be regulated primarily by HIF-1*α*, but increasing evidence indicates that HIF-2*α* is primarily responsible for hypoxia EPO induction [48–52]. Deletion of HIF-2*α*, but not HIF-1*α*, in adult mice gives rise to anemia, indicating its requirement for EPO regulation in physiologic and stress conditions [53]. In contrast, HIF-1*α* plays a critical role in regulation of hypoxic induction of vascular endothelial growth factor (VEGF) [54]. The *α*-subunit of HIF in the cytoplasm under normoxia is proline hydroxylated and ubiquitinated by the von Hippel-Lindau protein leading to rapid degradation by the proteasome. Hypoxia stabilizes the *α*-subunit of HIF that is then transported to the nucleus and heterodimerizes with the *β*-subunit for transactivation of target genes. Patients with genetic mutations in proline hydroxylase, von Hippel-Lindau protein, or HIF-2*α* have been identified and associated with familial erythrocytosis [55]. Regulation of EPO production by other transcription factors includes negative regulation by the GATA transcription factors [56] and activation by GATA-4 in fetal liver [57]. The Wilms tumor suppressor (Wt1) can upregulate EPO gene expression, and its colocalization with EPO in developing mice has led to the suggestion that Wt1 contributes to EPO gene regulation in hepatocytes and neuronal cells of the dorsal root ganglia [58].

## 5. Evidence of EPO Activity in Nonhematopoietic Tissue

Expression of the erythroid form of EpoR in nonhematopoietic tissues was first detected in endothelial cells providing for a mitogenic and chemotactic response to EPO [59, 60]. In mice, deletion of EPO (Epo^−/−^) or EpoR (EpoR^−/−^) leads to angiogenic defects detectable at day E10.5, two days prior to the onset of severe anemia [61]. Although EpoR expression is not HIF responsive, expression in endothelial cells can be induced in culture by the combination of EPO treatment and reduced oxygen tension [62, 63]. Interestingly, the endothelial response appears to contribute to the cardioprotective effects of EPO in animal models [64, 65]. Furthermore, EPO was found to enhance reendothelialization and prevent neointimal hyperplasia [66], and promote survival of primary human endothelial cells [67].

## 6. EpoR Expression in Brain

**Figure 2:**
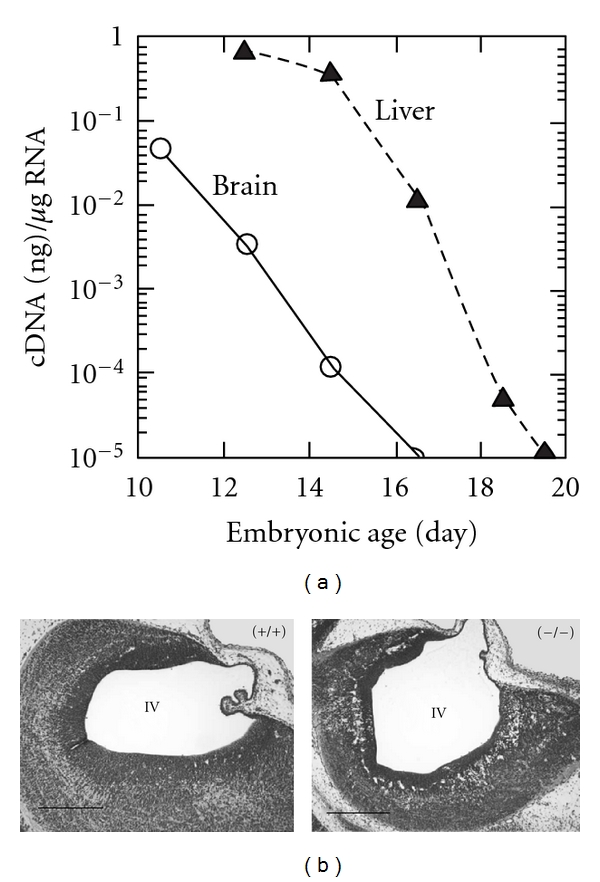
Erythropoietin receptor expression in mice during brain development. (a) Quantification of EpoR mRNA in mouse brain (circles) compared with liver (triangles) beginning at embryonic day E10 from Liu et al., [68]. (b) Hypoplasia of neuroepithelium of the fourth ventricle at embryonic day E12.5 in EpoR^−/−^ embryo (right) compared with EpoR^+/+^ embryo (left) (bars, 0.4 mm) from Yu et al., [69].

EpoR expression in neuronal cells, and the high level of EpoR expression in mouse brain midgestation, provided early evidence for potential EPO activity in brain (Figure 2(a)) [68, 70]. As with erythroid tissue, the extent of EpoR expression regulates EPO response in brain [71]. Mouse EpoR expression in the developing brain appears in regions associated with neurogenesis. For instance, EpoR localizes to the neural tube midgestation and is expressed at a high level comparable to adult hematopoietic tissue in mice but becomes subsequently downregulated by about 100-fold at birth [72]. EpoR expression and hence Epo signaling in brain decrease as development progresses in human. For instance, EpoR expression in adult brain is two orders of magnitude lower than the adult erythropoietic organ (bone marrow) [73]. Observations in the developing midbrain show EPO expression at day E11 in neurons attached to radial glial cells which transiently express EpoR [74]. At day E12.5, EpoR appears to shift to EPO expressing neurons adjacent to apoptotic bodies, and at day E14.5 apoptotic bodies appear without EPO expression in bands along the rostrocaudal length of the midbrain, collectively suggesting a role for EPO in brain morphogenesis. EPO expression in developing spinal cord and dorsal root ganglia follow EpoR expression in radial glial cells, providing more evidence that EPO may contribute to interaction among neurons and between neurons and radial glial cells, and promote differentiation or survival of specific subsets of neurons [75]. Both EPO and the small fraction of nonglycosylated EPO are downregulated during brain development. A heterodimer complex between the classical hematopoietic form of EpoR and the common *β* chain has been proposed as the receptor binding EPO in neural cells, but the common *β* chain does not appear to localize with EpoR or EPO in the rat brain and is below the level of detection in EPO responsive neuronal cell lines, SH-Sy5y and PC12 [76–78].

During the first two trimesters of human development, EpoR expression appears widespread [40]. In the developing embryo, EPO and EpoR expressions were detected in spinal cord and brain, as early as 7-8 weeks [37, 38]. At 20 weeks, EpoR expression in brain was localized on neurons, astrocytes, and choroids plexus. Meanwhile, EPO was also localized to neurons and astrocytes and expression of both EPO and EpoR persists after birth [36]. Lastly, EpoR expression is detected throughout the human, nonhuman primate and mouse brains, with EPO binding, particularly to areas of the hippocampus, capsula interna, cortex, and midbrain [33, 79].

## 7. EPO Is Neuroprotective

EPO stimulates proliferation of neural progenitor cells in culture. Moreover, the increased proliferation at modest hypoxia is mimicked by EPO treatment and is blocked by an EPO neutralizing antibody [69, 80–83]. Interestingly, EPO-stimulated proliferation in neural progenitor cultures is comparable to but less than FGF, and when added in combination does not increase proliferation beyond that of FGF alone [83]. Additionally, EPO was found to mimic in part the increase in dopaminergic neurons in day E12 rat mesencephalic precursor cells cultured at low oxygen tension [81]. Embryonic rat hippocampal and cortical neuron cultures demonstrate a protective effect of EPO from glutamate toxicity, and EPO promotes survival in the absence of trophic factors [69, 82]. EPO also provides protection to embryonic and postnatal hippocampal neurons from hypoxia-induced cell death [69, 84]. In the retina, EPO is inducible by HIF and is protective against light-induced damage [39], where hypoxia was found to increase EPO response and EpoR expression [69, 85].

In neural cells, EPO induces GATA-3, a GATA transcription family member that is required for brain development, which is able to transactivate the EpoR promoter [69, 86]; however, GATA-2 and GATA-4 may also contribute to EpoR expression in neural cells [87]. Brain-derived neurotrophic factor (BDNF) preconditioning of rat cortical cultures induced both EPO and sonic hedgehog, and both were required for BDNF neuroprotection [88]. Correspondingly, EPO treatment after ischemic stroke resulting in improved functional recovery induces BDNF and VEGF [89]. Spinal cord-derived neural progenitor cells also express EpoR that provides for EPO regulation of cell cycle and stimulation of proliferation [90].

## 8. Targeted Deletion of EpoR

Mice with targeted deletion of EpoR (EpoR^−/−^) exhibit increased apoptosis in the brain as early as day E10.5, thinning of the neuroepithelium, and smaller brain size prior to death around day E13.5 due to severe anemia (Figure 2(b)) [69, 74]. EpoR expression in neural progenitor cells is downregulated with differentiation and persists at a lower level in differentiated neurons [83]. A role for endogenous EPO in neuron maintenance and survival is suggested by cultures of neural cells lacking endogenous EpoR that exhibit reduced proliferative capacity and increased susceptibility to hypoxia and glutamate toxicity [69, 83]. A human EpoR transgene rescues the EpoR^−/−^ genotype, normalizes the erythroid potential and brain development, and rescues the increased apoptosis observed in the EpoR^−/−^ embryonic brain [91]. EpoR^−/−^ mice rescued with an erythroid restricted EpoR transgene survive to adulthood with normalized hematocrit and without any gross abnormal organ morphology [92]. However, adult mice that lack EpoR expression in the brain exhibit reduced neurogenesis in the subventricular zone and dentate gyrus and increased susceptibility to glutamate toxicity [83]. During ischemic stroke, mice that lack EpoR in neural cells show defective neural cell migration to the peri-infarct cortex [93]. Collectively, the increased apoptosis in embryonic brain, reduced neurogenesis in adult brain, and increased sensitivity to hypoxia and glutamate toxicity exhibited in mice with loss of EpoR expression in brain provide evidence that endogenous EPO signaling is neuroprotective throughout mouse development and adulthood.

## 9. EPO Signaling in Neuronal Cells

EPO stimulation of neuronal cells activates JAK2, STAT5, AKT, and MAPK signaling pathways [77, 94]. Increased EPO production in brain by transgene expression during ischemic injury resulted in activation of JAK2, ERK1/2, and AKT required for EPO-mediated neuroprotection [95, 96]. In hippocampal neurons, EPO was protective in free radical injury and maintained mitochondrial membrane potential [97]. The PI3K signaling pathway was also implicated in EPO protection to ischemic challenge [98]. Targeted deletion of STAT5 did not affect the EPO neuroprotective activity in hippocampal neurons but abrogated the EPO neurotrophic activity [99]. NF-*κ*B is associated with EPO neural protection but not with EPO erythroid activity (Figure 1) [100].

## 10. EPO and Neurovascular Response

**Figure 3:**
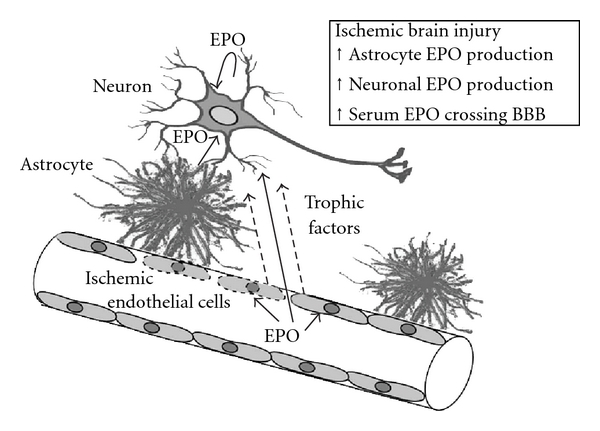
Ischemic brain injury and erythropoietin neuroprotection. EpoR expression by neurons mediates a direct neuroprotective EPO response. EPO production in astrocytes and neurons and EPO crossing the blood-brain barrier that is compromised during injury contribute to increased EPO in the local microenvironment. Endothelial cell EPO response can contribute indirectly to neuroprotection via improved oxygen delivery and secretion of neurotrophic factors.

Brain capillary endothelial cells express EpoR, where Epo acts directly as a competence factor [101]. EPO has been reported to regulate not only neurogenesis but also angiogenesis [80, 89]. Angiogenesis is a tightly controlled multistep process through which new blood vessels are formed by sprouting from the preexisting vasculature in the presence of VEGF and its receptor (VEGF-R). It is suggested that EPO may play a role in stimulating angiogenesis in response to ischemic injury in the brain possibly via VEGF upregulation [89]. Indeed, EpoR in microvascular/capillary endothelial cells is induced during evolution of cerebral infarct following permanent cerebral ischemia in mice and is further enhanced with EPO treatment [102, 103]. EPO treatment has been reported to have an antiapoptotic effect in cerebral vascular cells [104], and EPO-mediated neurovascular response has been suggested to occur via proangiogenic effects and through the regulation of cerebral blood flow. For instance, treatment with EPO was shown to upregulate EpoR expression in cerebral vascular endothelial cells, which in turn was suggested to drive neurovascular protection and angiogenesis and restore local cerebral blood flow in a mouse model of focal ischemia (Figure 3) [103].

## 11. Endothelial and Neuronal Cells Cross Talk

In the adult rodent brain, neuronal progenitor cells are localized adjacent to endothelial cells in the subventricular zone and the dentate gyrus [105]. Interestingly, cerebral ischemia has been reported to induce angiogenesis and neurogenesis [89, 106, 107]. Moreover, angiogenesis was shown to be coupled with neurogenesis [105, 108], whereby suppressing angiogenesis reduces neuroblast migration toward the ischemic cortex [109], thus indicating that interactive networks exist between endothelial and neuronal cells. The migration of new-born neurons toward the ischemic boundary region was found to be promoted by EPO-enhanced angiogenesis through matrix metalloproteinase-2 and -9 released by cerebral endothelial cells [110], thereby further highlighting the involvement of endothelial cell responses in regulation of neuronal cell responses. Neuronal progenitor cells can directly promote angiogenesis [111], and EPO treated neuronal progenitor cells induce angiogenesis through the production of VEGF and upregulation of VEGF-R2 in cerebral endothelial cells [112]. VEGF, in turn, was proposed to mediate neurogenesis by augmenting proliferation and neuronal differentiation of neural progenitor cells [106, 113].

## 12. Nitric Oxide (NO) and Neurovascular Response

Basal production of NO by endothelial nitric oxide synthase (eNOS) is believed to play pivotal roles in the regulation of cerebral blood flow, vascular tone, vascular resistance, and vascular growth under resting conditions in various mammals [114, 115]. In addition, vasodilation is suppressed with specific inhibition of neuronal NOS (nNOS) and in mice that lack nNOS [116, 117]. Interestingly, EPO has been reported to exert neuroprotective effects in vivo, by regulating neurovascular response resulting in vasodilation and increased blood flow through increasing NO production [118]. It is noteworthy that NO can induce EpoR expression in neuronal cell cultures [119], can improve O_2_ supply by means of vasodilation, and thus can provide tissue protective effects, although excessive production of NO is neurotoxic [120]. Overproduction of NO by inducible NOS (iNOS) during inflammation has been implicated in various pathological processes, including tissue injury and cell apoptosis caused by ischemia and inflammation [121, 122]. Separate eNOS-, nNOS- or iNOS-deficient mouse models have been useful in demonstrating that the eNOS isoform is protective against focal cerebral ischemic injury, while the nNOS and the iNOS isoforms play roles in early and later stages of ischemic injury, respectively [122–124]. nNOS isoform was found to be heavily involved in hemodynamic response to local neuronal activity, a process called neurovascular coupling [116]. In relation to the neuroprotective effects of EPO, using primary dorsal root ganglion cultures, NO administration was found to protect against axonal degeneration in a manner dependent on HIF-mediated transcription of EPO in glial cells [125]. These findings collectively underscore the link between the EPO responses in brain and NO.

## 13. EPO Neuroprotection in Animal Models

Studies using animal models provide ample evidence for the neuroprotective activity of EPO. EPO stimulation of neural and endothelial cells and EPO production in brain have been suggested to contribute to neuroprotection, with the latter being of particular importance since only low levels of EPO appear to cross the blood-brain barrier when administered at high dose intravenously [126]. EPO has been reported to inhibit neuronal cell apoptosis, stimulate cell survival and differentiation, and promote neurogenesis and neurotrophic functions. To date, EPO-mediated neuroprotection has been shown to occur via (i) antiapoptotic response in neurons, (ii) endothelial response by increased blood flow and oxygen delivery through increased vascular relaxation and angiogenesis, and (iii) anti-inflammatory effects.

In gerbils, exogenous EPO administered directly to the brain was neuroprotective against brain ischemia, while infusion of soluble EpoR increased susceptibility to ischemia, and mild ischemia resulted in neural degeneration and impaired learning [127]. In a rat model of ischemic stroke, direct infusion of EPO in brain was neuroprotective and improved performance in the Morris water maze [128, 129]. In mice, EpoR expression increased with ischemic stroke along with increased EPO production, and EPO treatment reduced infarct size [102], suggesting that in ischemic injury, EpoR increases EPO response and the increase of EPO availability contributes to neuroprotection. The neuroprotective effect of intraperitoneally administered EPO in traumatic brain injury improved mitochondrial function and was demonstrated to be independent of hematocrit by hemodilution [130, 131]. Cross talk between EPO and other cytokines including the requirement for TNF receptor contributes to EPO neuroprotection [132]. In addition to its effects on neuronal cells, recent evidence suggests that EPO also protects nonneuronal cells, namely, oligodendrocytes and astrocytes via inhibiting apoptosis and promoting survival [133], and possibly preventing long-term brain damage by inhibiting glial and astroglial cell swelling [134, 135].

In hypoxic preconditioning when mild brain ischemia protects against severe ischemic challenge, HIF activation increases VEGF and EPO expression to promote tolerance to brain ischemia [129]. Blocking EPO signaling by direct infusion of soluble EpoR in the brain blocked hypoxic preconditioning [94, 136, 137]. EPO activity in hypoxic preconditioning increases survival of neutrally differentiated embryonic stem cells following transplantation in the ischemic rat brain [138]. Activation of HIF in neurons by ischemia, iron chelators, or agents that stabilize HIF increases expression of HIF-regulated genes, such as EPO, glycolytic enzymes, and p21, and provides neuroprotection in hypoxic or oxidative stress [139–141]. Knockdown of HIF-1*α* in astrocytes decreased VEGF but not EPO expression, and targeted deletion of HIF-1*α* in neural cells in mice reduced VEGF but not EPO [50, 142]. In contrast, knockdown of HIF-2*α* in astrocytes abrogated hypoxic induction of EPO, consistent with the specificity of HIF-2*α* in EPO regulation, which was also indicated by the postnatal targeted deletion of HIF-2*α* in mice leading to anemia [50, 53]. EPO also contributes to hypoxic post-conditioning in a mouse model of cerebral ischemia [143].

## 14. EPO Activity in Neonatal Rodent Models

Direct brain injection of EPO provided protection in a neonatal rat model of hypoxic-ischemic injury and reduced the size and extent of brain damage [144]. Pretreatment with intraperitoneally injected EPO in neonatal mice was also neuroprotective in hypoxic-ischemic injury [145]. In neonatal rats, EPO neuroprotection was found to provide a long-term effect in the developing brain that was greater in females than in males [146]. EPO treatment in neonatal hypoxic-ischemic brain injury in rats increased brain weight, decreased apoptosis, protected dopamine neurons, and improved functional outcomes including long-term spatial memory deficits [147–150]. EPO administration in ischemic injury in the developing brain favored neurogenesis and decreased gliogenesis [151]. EPO also improved auditory processing and sensorimotor function in hypoxia-ischemic injury [152, 153].

Neonatal ischemic brain injury in rats induces EpoR expression in the ischemic area in neurons and microglia/macrophage [154, 155]. In neonatal rodents subjected to hyperoxia, exogenous EPO reduced apoptosis, caspase activity, and proteome changes in brain [156, 157]. Protection from brain ischemic injury by EPO treatment in neonatal mice decreased expression of proinflammatory genes [158, 159]. Inflammatory response and white matter damage in postnatal rats subjected to lipopolysaccharide-induced injury or *E. coli* infection in utero are also decreased by EPO treatment [160, 161]. Interestingly, EPO neuroprotection is dose dependent and EPO treatment was found to be ineffective at low dose and at multiple high doses [162].

In a model for human early-third trimester placental insufficiency, transient systemic hypoxia-ischemia on day E18 rats demonstrated EpoR increased on oligodendroglial cells and neurons, and EPO treatment postnatally protected oligodendrocytes and neurons, minimized histological damage, and improved motor skill performance in adults [163]. In hypoxic/ischemic injury in neonatal and adult rats, delayed EPO treatment beginning 6 hours after injury reduced lesion volume while treatment beginning 24 to 48 hr after injury did not affect infarct volume but improved oligodendrogenesis and white matter restoration, neurogenesis and precursor migration, and neurological function at 2 to 6 weeks after injury [164, 165]. The neuroprotective effects observed in cell culture and in animal models suggested the potential of high-dose EPO therapy to protect against brain injury in extremely low-birth-weight infants [166, 167].

## 15. EPO and Inflammation

 Treatment with recombinant human EPO has been reported to stimulate anti-inflammatory signaling, which was suggested to contribute to its direct neuroprotective effect during cerebral ischemia [168]. Inflammation plays a critical role in the pathogenesis of cerebral ischemia, where the influx of leukocytes from the blood into the brain and activation of resident microglial cells to secrete inflammatory mediators and cytokines result in barrier damage, microvascular occlusion, and, thus, aggravate injury [169, 170]. The administration of EPO to animals with experimental cerebral ischemia resulted in the reduction of the local production of TNF-*α*, IL-6, and the chemokine MCP-1, subsequently leading to a marked reduction of infarct size [168]. Although EPO-mediated reduction of ischemia-induced inflammation has been proposed to occur via reducing neuronal death rather than by direct effects upon EpoR-expressing inflammatory cells [168], it remains unknown whether or not EPO can play a direct role in regulating inflammatory cell responses. In an experimental model of multiple sclerosis, an autoimmune disease of the CNS, administration of EPO inhibited the inflammatory response, delayed the onset of the disease, and decreased its severity [171–174]. Similarly, in a rat model of optic neuritis, systemic administration of EPO significantly increased the survival of retinal ganglion cells [175]. Although the exact mechanisms underlying the anti-inflammatory effects of EPO remain unknown, EPO might act by reducing leukocyte transmigration through endothelial cells, since it enhances the resistance of endothelial cells toward ischemia [104] and could be mediated by activating immune suppressive lymphocyte response [174]. In addition, EPO-mediated oligodendrogenesis and the inhibition of oligodendrocyte cytotoxicity, induced by inflammatory stimuli, could also contribute to the observed neuroprotection in experimental multiple sclerosis [171, 176].

## 16. EPO and Retinopathy

As previously mentioned, the neuroprotective effect of EPO extends to the retina and provides protection to mouse photoreceptor cells against UV light damage [39]. During human development, EPO mRNA in the retina and EPO protein in the vitreous at levels greater than in serum were observed between 12 and 24 weeks gestation and increased with gestational age [177]. However, concerns about the angiogenic properties of EPO remain. Association between elevated EPO in the vitreous and proliferative retinopathy was observed in adult diabetic patients exhibiting increased levels of VEGF and EPO in the vitreous fluid compared with nondiabetic patients with ocular disease [178]. EPO was found to be more strongly associated with diabetic retinopathy than VEGF, and the level of EPO in the vitreous did not correlate with plasma level, suggesting local production of EPO. Indeed, a single nucleotide polymorphism in the human EPO gene promoter is associated with increased EPO in the vitreous and severe microvascular complications in diabetes, proliferative diabetic retinopathy, and end-stage renal disease [179].

In animal studies, mice with a green fluorescent protein transgene driven by EPO regulatory sequences confirm EPO production in the ganglion cell layer in the developing retina coincident with newborn-associated anemia [180]. In a murine model of retinopathy related to prematurity, neovascularization that was induced on the vitreous side of the inner limiting membrane by hyperoxia-normoxia was absent in mice with knockdown of HIF-2*α* and these mice exhibited degeneration of neural layers in the retina with subsequent prolonged normoxia and a marked attenuation of EPO expression [52]. The loss of EPO expression and associated degeneration of neural layers relate to the induction of EPO by hypoxia preconditioning and resultant protection of photoreceptor cells in the retina [39]. This protective effect is mimicked by constitutive overexpression of EPO in the retina [181]. The protective effect of EPO in the retina appears to be dependent on the timing and extent of treatment [182]. For instance, in a mouse model of retinopathy, early EPO administration provided protection to hypoxia-induced retinal neuron apoptosis, while late administration enhanced pathological revascularization [182].

## 17. EPO and Stroke

Attention was drawn to the potential for EPO therapy in neurological diseases by the initial pilot study of 40 adult stroke patients that suggested high dose EPO administration was well tolerated in acute ischemic stroke and was associated with improved clinical outcome [183]. Subsequently, an expanded trial of 460 patients revealed that a high number of stroke patients received tissue plasminogen activator (TPA) and that there was a significant increase in overall death rate with EPO treatment (42/256) compared with the placebo group (24/266) [184]. Further animal studies revealed that in rodents treated with TPA after middle cerebral artery occlusion, EPO administered up to 6 hours after reperfusion showed no reduction in the volume of ischemic injury and a significant increase in the incidence of brain hemorrhage. Moreover, the expected EPO-associated reduction in brain swelling was abolished with TPA, underscoring again the importance of timing in EPO therapy [185, 186]. Of note, delayed EPO treatment was neuroprotective in a rat model of traumatic brain injury [187].

 It is noteworthy that clinical trials using EPO to treat disease associated anemia to achieve a high hemoglobin level have demonstrated adverse effects associated with EPO therapy. These include efforts to improve clinical outcome and reduce cardiovascular events in patients with congestive heart failure or ischemic heart disease undergoing dialysis, patients with anemia of chronic kidney disease not undergoing dialysis, and those with type 2 diabetes mellitus [188–190]. In the latter trial [189], higher risks of cardiovascular events or death were associated with the subgroup of patients with poor initial hematopoietic response to EPO treatment [191]. In cancer patients, EPO treatment to increase hemoglobin levels approaching the normal range resulted in increased adverse events including venous thromboembolism and cancer progression, particularly in select solid cancers such as metastatic breast cancer and head and neck cancer [192–194].

 With increasing awareness of the potential adverse effects associated with high dose EPO treatment, investigations of EPO therapy for neurological disorders such as stroke and schizophrenia are on-going with consideration given to exclusion of treatment with TPA, EPO modifications to minimize changes in hematocrit, and combination therapy with other hormones [195–199].

## 18. EPO, Hypothalamus, and Metabolic Regulation

Recently, several metabolic effects for EPO have been described. For example, EPO treatment in obese mice decreased body weight, fat mass and blood glucose in a dose-dependent manner [200–203] and provided protection in mouse models of type 1 and type 2 diabetes [204]. These EPO activities were attributed to EPO response in multiple tissues including pancreatic beta cells, skeletal muscle, adipose tissue and brain, independent of changes in hematocrit [200, 203, 204]. Indeed, the significant decrease in food consumption in response to high-dose EPO treatment in association with the decreased body weight and fat mass in obese animals correlated with EPO activity in the hypothalamus [200, 201]. EpoR expression in hypothalamus was found to localize to proopiomelanocortin (POMC) neurons that regulate food intake, and EPO treatment increased POMC production in the hypothalamus [200]. Conversely, no increase in POMC production or decrease in fat mass was observed with EPO treatment in mice with EPO signaling restricted to erythroid tissue that exhibit an obese phenotype [200]. These observations suggest a direct link between EPO and POMC neurons in the hypothalamus during energy and metabolic regulation.

## 19. Conclusion

Identification of the spatial and temporal expression of EPO and EpoR during brain development has facilitated understanding of the potential range of EPO response in brain during ischemic/hypoxic stress or disease and provided insight on the utility of EPO treatment. The pleiotropic activity of EPO evident in culture and animal studies remain of continuing interest, particularly its potential treatment for neurological disease. EpoR expression in neuronal and endothelial cells and hypoxic-induction of EPO production in brain likely drive the cytoprotective outcome, through antiapoptotic, vascular, and anti-inflammatory responses. In addition, the hypoxic induction of EPO in the kidney drives the erythroid response to increase blood cell production in the bone marrow leading to increased oxygen delivery and neuroprotection. Contributing to increased oxygen delivery is endothelial response to EPO, which leads to elevated NO production, vasodilation, and increased blood flow. In conclusion, the high level of EpoR expression in the developing brain including neural progenitor cells and neurons, and continued EpoR expression in adult brain can support direct proliferative and antiapoptotic responses to EPO, suggesting that local EPO production and/or availability can directly promote a neuroprotective response involving neural and endothelial cells collaboration to promote the brain response to EPO (see Figure 3).

## Abbreviations

EPO: Erythropoietin
EpoR: Erythropoietin receptor
JAK: Janus kinase
CFU-E: Colony forming unit-erythroid
HIF: Hypoxia-inducible factor
CNS: Central nervous system
HER: Hypoxia-responsive element
Wt1: Wilms tumor suppressor
BDNF: Brain-derived neurotrophic factor
VEGF: Vascular endothelial growth factor
VEGF-R: VEGF receptor
NO: Nitric oxide
NOS: NO synthase
TPA: Tissue plasminogen activator
POMC: Proopiomelanocortin.

## References

[1] Lin F-K, Suggs S, Lin C-H (1985). Cloning and expression of the human erythropoietin gene. *Proceedings of the National Academy of Sciences of the United States of America*.

[2] Jacobs K, Shoemaker C, Rudersdorf R (1985). Isolation and characterization of genomic and cDNA clones of human erythropoietin. *Nature*.

[3] Lai P-H, Everett R, Wang F-F (1986). Structural characterization of human erythropoietin. *Journal of Biological Chemistry*.

[4] Sasaki H, Bothner B, Dell A, Fukuda M (1987). Carbohydrate structure of erythropoietin expressed in Chinese hamster ovary cells by a human erythropoietin cDNA. *Journal of Biological Chemistry*.

[5] Fukuda MN, Sasaki H, Lopez L, Fukuda M (1989). Survival of recombinant erythropoietin in the circulation: the role of carbohydrates. *Blood*.

[6] D’Andrea AD, Zhu Y (1996). Cloning and functional analysis of erythropoietin-, interleukin-3-and thrombopoietin-inducible genes. *Stem Cells*.

[7] Wen D, Boissel JP, Showers M, Ruch BC, Bunn HF (1994). Erythropoietin structure-function relationships. Identification of functionally important domains. *Journal of Biological Chemistry*.

[8] Parganas E, Wang D, Stravopodis D (1998). Jak2 is essential for signaling through a variety of cytokine receptors. *Cell*.

[9] Syed RS, Reid SW, Li C (1998). Efficiency of signalling through cytokine receptors depends critically on receptor orientation. *Nature*.

[10] Noguchi CT, Bae KS, Chin K, Wada Y, Schechter AN, Hankins WD (1991). Cloning of the human erythropoietin receptor gene. *Blood*.

[11] Youssoufian H, Longmore G, Neumann D, Yoshimura A, Lodish HF (1993). Structure, function, and activation of the erythropoietin receptor. *Blood*.

[12] Funakoshi-Tago M, Pelletier S, Moritake H, Parganas E, Ihle JN (2008). Jak2 FERM domain interaction with the erythropoietin receptor regulates Jak2 kinase activity. *Molecular and Cellular Biology*.

[13] Livnah O, Stura EA, Middleton SA, Johnson DL, Jolliffe LK, Wilson IA (1999). Crystallographic evidence for preformed dimers of erythropoietin receptor before ligand activation. *Science*.

[14] Wu H, Liu X, Jaenisch R, Lodish HF (1995). Generation of committed erythroid BFU-E and CFU-E progenitors does not require erythropoietin or the erythropoietin receptor. *Cell*.

[15] Lin CS, Lim SK, D’Agati V, Costantini F (1996). Differential effects of an erythropoietin receptor gene disruption on primitive and definitive erytnropoiesis. *Genes and Development*.

[16] Palis J, McGrath KE, Kingsley PD (1995). Initiation of hematopoiesis and vasculogenesis in murine yolk sac explants. *Blood*.

[17] Peeters M, Ottersbach K, Bollerot K (2009). Ventral embryonic tissues and Hedgehog proteins induce early AGM hematopoietic stem cell development. *Development*.

[18] Lee R, Kertesz N, Joseph SB, Jegalian A, Wu H (2001). Erythropoietin (Epo) and EpoR expression and 2 waves of erythropoiesis. *Blood*.

[19] McGann JK, Silver L, Liesveld J, Palis J (1997). Erythropoietin-receptor expression and function during the initiation of murine yolk sac erythropoiesis. *Experimental Hematology*.

[20] Zimmermann F, Rich IN (1997). Mammalian homeobox B6 expression can be correlated with erythropoietin production sites and erythropoiesis during development, but not with hematopoietic or nonhematopoietic stem cell populations. *Blood*.

[21] Obara N, Suzuki N, Kim K, Nagasawa T, Imagawa S, Yamamoto M (2008). Repression via the GATA box is essential for tissue-specific erythropoietin gene expression. *Blood*.

[22] Semenza GL, Koury ST, Nejfelt MK, Gearhart JD, Antonarakis SE (1991). Cell-type-specific and hypoxia-inducible expression of the human erythropoietin gene in transgenic mice. *Proceedings of the National Academy of Sciences of the United States of America*.

[23] Koury ST, Bondurant MC, Koury MJ (1988). Localization of erythropoietin synthesizing cells in murine kidneys by in situ hybridization. *Blood*.

[24] Maxwell PH, Osmond MK, Pugh CW (1993). Identification of the renal erythropoietin-producing cells using transgenic mice. *Kidney International*.

[25] Tan CC, Eckardt K, Ratcliffe PJ (1991). Organ distribution of erythropoietin messenger RNA in normal and uremic rats. *Kidney International*.

[26] Fandrey J, Bunn HF (1993). In vivo and in vitro regulation of erythropoietin mRNA: measurement by competitive polymerase chain reaction. *Blood*.

[27] Köchling J, Curtin PT, Madan A (1998). Regulation of human erythropoietin gene induction by upstream flanking sequences in transgenic mice. *British Journal of Haematology*.

[28] Koury ST, Bondurant MC, Koury MJ, Semenza GL (1991). Localization of cells producing erythropoietin in murine liver by in situ hybridization. *Blood*.

[29] Yasuda Y, Masuda S, Chikuma M, Inoue K, Nagao M, Sasaki R (1998). Estrogen-dependent production of erythropoietin in uterus and its implication in uterine angiogenesis. *Journal of Biological Chemistry*.

[30] Chikuma M, Masuda S, Kobayashi T, Nagao M, Sasaki R (2000). Tissue-specific regulation of erythropoietin production in the murine kidney, brain, and uterus. *American Journal of Physiology*.

[31] Magnanti M, Gandini O, Giuliani L (2001). Erythropoietin expression in primary rat Sertoli and peritubular myoid cells. *Blood*.

[32] Kobayashi T, Yanase H, Iwanaga T, Sasaki R, Nagao M (2002). Epididymis is a novel site of erythropoietin production in mouse reproductive organs. *Biochemical and Biophysical Research Communications*.

[33] Marti HH (1996). Erythropoietin gene expression in human, monkey and murine brain. *European Journal of Neuroscience*.

[34] Masuda S, Okano M, Yamagishi K, Nagao M, Ueda M, Sasaki R (1994). A novel site of erythropoietin production. Oxygen-dependent production in cultured rat astrocytes. *Journal of Biological Chemistry*.

[35] Dame C, Bartmann P, Wolber EM, Fahnenstich H, Hofmann D, Fandrey J (2000). Erythropoietin gene expression in different areas of the developing human central nervous system. *Developmental Brain Research*.

[36] Juul SE, Yachnis AT, Rojiani AM, Christensen RD (1999). Immunohistochemical localization of erythropoietin and its receptor in the developing human brain. *Pediatric and Developmental Pathology*.

[37] Li Y, Juul SE, Morris-Wiman JA, Calhoun DA, Christensen RD (1996). Erythropoietin receptors are expressed in the central nervous system of mid-trimester human fetuses. *Pediatric Research*.

[38] Juul SE, Anderson DK, Li Y, Christensen RD (1998). Erythropoietin and erythropoietin receptor in the developing human central nervous system. *Pediatric Research*.

[39] Grimm C, Wenzel A, Groszer M (2002). HIF-1-induced erythropoietin in the hypoxic retina protects against light-induced retinal degeneration. *Nature Medicine*.

[40] Juul SE, Yachnis AT, Christensen RD (1998). Tissue distribution of erythropoietin and erythropoietin receptor in the developing human fetus. *Early Human Development*.

[41] Juul SE, Harcum J, Li Y, Christensen RD (1997). Erythropoietin is present in the cerebrospinal fluid of neonates. *Journal of Pediatrics*.

[42] Beck I, Ramirez S, Weinmann R, Caro J (1991). Enhancer element at the 3’-flanking region controls transcriptional response to hypoxia in the human erythropoietin gene. *Journal of Biological Chemistry*.

[43] Semenza GL, Nejfelt MK, Chi SM, Antonarakis SE (1991). Hypoxia-inducible nuclear factors bind to an enhancer element located 3′ to the human erythropoietin gene. *Proceedings of the National Academy of Sciences of the United States of America*.

[44] Blanchard KL, Acquaviva AM, Galson DL, Bunn HF (1992). Hypoxic induction of the human erythropoietin gene: cooperation between the promoter and enhancer, each of which contains steroid receptor response elements. *Molecular and Cellular Biology*.

[45] Bunn HF, Gu J, Huang LE, Park JW, Zhu H (1998). Erythropoietin: a model system for studying oxygen-dependent gene regulation. *Journal of Experimental Biology*.

[46] Smith TG, Robbins PA, Ratcliffe PJ (2008). The human side of hypoxia-inducible factor. *British Journal of Haematology*.

[47] Metzen E, Ratcliffe PJ (2004). HIF hydroxylation and cellular oxygen sensing. *Biological Chemistry*.

[48] Percy MJ, Furlow PW, Lucas GS (2008). A gain-of-function mutation in the HIF2A gene in familial erythrocytosis. *New England Journal of Medicine*.

[49] Rankin EB, Biju MP, Liu Q (2007). Hypoxia-inducible factor-2 (HIF-2) regulates hepatic erythropoietin in vivo. *Journal of Clinical Investigation*.

[50] Chavez JC, Baranova O, Lin J, Pichiule P (2006). The transcriptional activator hypoxia inducible factor 2 (HIF-2/EPAS-1) regulates the oxygen-dependent expression of erythropoietin in cortical astrocytes. *Journal of Neuroscience*.

[51] Warnecke C, Zaborowska Z, Kurreck J (2004). Differentiating the functional role of hypoxia-inducible factor (HIF)-1*α* and HIF-2*α* (EPAS-1) by the use of RNA interference: erythropoietin is a HIF-2*α* target gene in Hep3B and Kelly cells. *FASEB Journal*.

[52] Morita M, Ohneda O, Yamashita T (2003). HLF/HIF-2*α* is a key factor in retinopathy of prematurity in association with erythropoietin. *EMBO Journal*.

[53] Gruber M, Hu CJ, Johnson RS, Brown EJ, Keith B, Simon MC (2007). Acute postnatal ablation of Hif-2*α* results in anemia. *Proceedings of the National Academy of Sciences of the United States of America*.

[54] Tang N, Wang L, Esko J (2004). Loss of HIF-1*α* in endothelial cells disrupts a hypoxia-driven VEGF autocrine loop necessary for tumorigenesis. *Cancer Cell*.

[55] Semenza GL (2009). Involvement of oxygen-sensing pathways in physiologic and pathologic erythropoiesis. *Blood*.

[56] Imagawa S, Yamamoto M, Miura Y (1997). Negative regulation of the erythropoietin gene expression by the GATA transcription factors. *Blood*.

[57] Dame C, Sola MC, Lim KC (2004). Hepatic erythropoietin gene regulation by GATA-4. *Journal of Biological Chemistry*.

[58] Dame C, Kirschner KM, Bartz KV, Wallach T, Hussels CS, Scholz H (2006). Wilms tumor suppressor, Wt1, is a transcriptional activator of the erythropoietin gene. *Blood*.

[59] Anagnostou A, Lee ES, Kessimian N, Levinson R, Steiner M (1990). Erythropoietin has a mitogenic and positive chemotactic effect on endothelial cells. *Proceedings of the National Academy of Sciences of the United States of America*.

[60] Anagnostou A, Liu Z, Steiner M (1994). Erythropoietin receptor mRNA expression in human endothelial cells. *Proceedings of the National Academy of Sciences of the United States of America*.

[61] Kertesz N, Wu J, Chen THP, Sucov HM, Wu H (2004). The role of erythropoietin in regulating angiogenesis. *Developmental Biology*.

[62] Beleslin-Cokic BB, Cokic VP, Yu X, Weksler BB, Schechter AN, Noguchi CT (2004). Erythropoietin and hypoxia stimulate erythropoietin receptor and nitric oxide production by endothelial cells. *Blood*.

[63] Beleslin-Čokić BB, Čokić VP, Wang L (2011). Erythropoietin and hypoxia increase erythropoietin receptor and nitric oxide levels in lung microvascular endothelial cells. *Cytokine*.

[64] Westenbrink BD, Lipšic E, Van Der Meer P (2007). Erythropoietin improves cardiac function through endothelial progenitor cell and vascular endothelial growth factor mediated neovascularization. *European Heart Journal*.

[65] Teng R, Calvert JW, Sibmooh N (2011). Acute erythropoietin cardioprotection is mediated by endothelial response. *Basic Research in Cardiology*.

[66] Urao N, Okigaki M, Yamada H (2006). Erythropoietin-mobilized endothelial progenitors enhance reendothelialization via Akt-endothelial nitric oxide synthase activation and prevent neointimal hyperplasia. *Circulation Research*.

[67] Zhande R, Karsan A (2007). Erythropoietin promotes survival of primary human endothelial cells through PI3K-dependent, NF-*κ*B-independent upregulation of Bcl-xL. *American Journal of Physiology*.

[68] Liu ZY, Chin K, Noguchi CT (1994). Tissue specific expression of human erythropoietin receptor in transgenic mice. *Developmental Biology*.

[69] Yu X, Shacka JJ, Eells JB (2002). Erythropoietin receptor signalling is required for normal brain development. *Development*.

[70] Masuda S, Nagao M, Takahata K (1993). Functional erythropoietin receptor of the cells with neural characteristics. Comparison with receptor properties of erythroid cells. *Journal of Biological Chemistry*.

[71] Sanchez PE, Fares RP, Risso JJ (2009). Optimal neuroprotection by erythropoietin requires elevated expression of its receptor in neurons. *Proceedings of the National Academy of Sciences of the United States of America*.

[72] Liu C, Shen K, Liu Z, Noguchi CT (1997). Regulated human erythropoietin receptor expression in mouse brain. *Journal of Biological Chemistry*.

[73] Chin K, Oda N, Shen K, Noguchi CT (1995). Regulation of transcription of the human erythropoietin receptor gene by proteins binding to GATA-1 and Sp1 motifs. *Nucleic Acids Research*.

[74] Knabe W, Knerlich F, Washausen S (2004). Expression patterns of erythropoietin and its receptor in the developing midbrain. *Anatomy and Embryology*.

[75] Knabe W, Sirén AL, Ehrenreich H, Kuhn HJ (2005). Expression patterns of erythropoietin and its receptor in the developing spinal cord and dorsal root ganglia. *Anatomy and Embryology*.

[76] Brines M, Grasso G, Fiordaliso F (2004). Erythropoietin mediates tissue protection through an erythropoietin and common *β*-subunit heteroreceptor. *Proceedings of the National Academy of Sciences of the United States of America*.

[77] Um M, Gross AW, Lodish HF (2007). A "classical" homodimeric erythropoietin receptor is essential for the antiapoptotic effects of erythropoietin on differentiated neuroblastoma SH-SY5Y and pheochromocytoma PC-12 cells. *Cellular Signalling*.

[78] Sanchez PE, Navarro FP, Fares RP (2009). Erythropoietin receptor expression is concordant with erythropoietin but not with common *β* chain expression in the rat brain throughout the life span. *Journal of Comparative Neurology*.

[79] Digicaylioglu M, Bichet S, Marti HH (1995). Localization of specific erythropoietin binding sites in defined areas of the mouse brain. *Proceedings of the National Academy of Sciences of the United States of America*.

[80] Shingo T, Todd Sorokan S, Shimazaki T, Weiss S (2001). Erythropoietin regulates the in vitro and in vivo production of neuronal progenitors by mammalian forebrain neural stem cells. *Journal of Neuroscience*.

[81] Studer L, Csete M, Lee SH (2000). Enhanced proliferation, survival, and dopaminergic differentiation of CNS precursors in lowered oxygen. *Journal of Neuroscience*.

[82] Morishita E, Masuda S, Nagao M, Yasuda Y, Sasaki R (1996). Erythropoetin receptor is expressed in rat hippocampal and cerebral cortical neurons, and erythropoietin prevents in vitro glutamate-induced neuronal death. *Neuroscience*.

[83] Chen ZY, Asavaritikrai P, Prchal JT, Noguchi CT (2007). Endogenous erythropoietin signaling is required for normal neural progenitor cell proliferation. *Journal of Biological Chemistry*.

[84] Lewczuk P, Hasselblatt M, Kamrowski-Kruck H (2000). Survival of hippocampal neurons in culture upon hypoxia: effect of erythropoietin. *NeuroReport*.

[85] Chin K, Yu X, Beleslin-Cokic B (2000). Production and processing of erythropoietin receptor transcripts in brain. *Molecular Brain Research*.

[86] Pandolfi PP, Roth ME, Karis A (1995). Targeted disruption of the GATA3 gene causes severe abnormalities in the nervous system and in fetal liver haematopoiesis. *Nature Genetics*.

[87] Wallach I, Zhang J, Hartmann A (2009). Erythropoietin-receptor gene regulation in neuronal cells. *Pediatric Research*.

[88] Wu CL, Chen SD, Yin JH, Hwang CS, Yang DI (2010). Erythropoietin and sonic hedgehog mediate the neuroprotective effects of brain-derived neurotrophic factor against mitochondrial inhibition. *Neurobiology of Disease*.

[89] Wang L, Zhang Z, Wang Y, Zhang R, Chopp M (2004). Treatment of stroke with erythropoietin enhances neurogenesis and angiogenesis and improves neurological function in rats. *Stroke*.

[90] Wang Y, Yao M, Zhou C (2010). Erythropoietin promotes spinal cord-derived neural progenitor cell proliferation by regulating cell cycle. *Neuroscience*.

[91] Yu X, Lin CS, Costantini F, Noguchi CT (2001). The human erythropoietin receptor gene rescues erythropoiesis and developmental defects in the erythropoietin receptor null mouse. *Blood*.

[92] Suzuki N, Ohneda O, Takahashi S (2002). Erythroid-specific expression of the erythropoietin receptor rescued its null mutant mice from lethality. *Blood*.

[93] Tsai PT, Ohab JJ, Kertesz N (2006). A critical role of erythropoietin receptor in neurogenesis and post-stroke recovery. *Journal of Neuroscience*.

[94] Ruscher K, Freyer D, Karsch M (2002). Erythropoietin is a paracrine mediator of ischemic tolerance in the brain: evidence from an in vitro model. *Journal of Neuroscience*.

[95] Kilic E, Kilic Ü, Soliz J, Bassetti CL, Gassmaim M, Hermann DM (2005). Brain-derived erythropoietin protects from focal cerebral ischemia by dual activation of ERK-1/-2 and Akt pathways. *FASEB Journal*.

[96] Sola A, Rogido M, Lee BH, Genetta T, Wen TC (2005). Erythropoietin after focal cerebral ischemia activates the Janus kinase-signal transducer and activator of transcription signaling pathway and improves brain injury in postnatal day 7 rats. *Pediatric Research*.

[97] Chong ZZ, Kang JQ, Maiese K (2003). Erythropoietin fosters both intrinsic and extrinsic neuronal protection through modulation of microglia, Akt1, Bad, and caspase-mediated pathways. *British Journal of Pharmacology*.

[98] Zhang F, Signore AP, Zhou Z, Wang S, Cao G, Chen J (2006). Erythropoietin protects CA1 neurons against global cerebral ischemia in rat: potential signaling mechanisms. *Journal of Neuroscience Research*.

[99] Byts N, Samoylenko A, Fasshauer T (2008). Essential role for Stat5 in the neurotrophic but not in the neuroprotective effect of erythropoietin. *Cell Death and Differentiation*.

[100] Digicaylioglu M, Lipton SA (2001). Erythropoietin-mediated neuroprotection involves cross-talk between Jak2 and NF-*κ*B signalling cascades. *Nature*.

[101] Yamaji R, Okada T, Moriya M (1996). Brain capillary endothelial cells express two forms of erythropoietin receptor mRNA. *European Journal of Biochemistry*.

[102] Bernaudin M, Marti HH, Roussel S (1999). A potential role for erythropoietin in focal permanent cerebral ischemia in mice. *Journal of Cerebral Blood Flow and Metabolism*.

[103] Li Y, Lu Z, Keogh CL, Yu SP, Wei L (2007). Erythropoietin-induced neurovascular protection, angiogenesis, and cerebral blood flow restoration after focal ischemia in mice. *Journal of Cerebral Blood Flow and Metabolism*.

[104] Chong ZZ, Kang JQ, Maiese K (2002). Erythropoietin is a novel vascular protectant through activation of AKt1 and mitochondrial modulation of cysteine proteases. *Circulation*.

[105] Palmer TD, Willhoite AR, Gage FH (2000). Vascular niche for adult hippocampal neurogenesis. *Journal of Comparative Neurology*.

[106] Jin K, Minami M, Lan JQ (2001). Neurogenesis in dentate subgranular zone and rostral subventricular zone after focal cerebral ischemia in the rat. *Proceedings of the National Academy of Sciences of the United States of America*.

[107] Arvidsson A, Collin T, Kirik D, Kokaia Z, Lindvall O (2002). Neuronal replacement from endogenous precursors in the adult brain after stroke. *Nature Medicine*.

[108] Louissaint A, Rao S, Leventhal C, Goldman SA (2002). Coordinated interaction of neurogenesis and angiogenesis in the adult songbird brain. *Neuron*.

[109] Ohab JJ, Fleming S, Blesch A, Carmichael ST (2006). A neurovascular niche for neurogenesis after stroke. *Journal of Neuroscience*.

[110] Wang L, Zheng GZ, Rui LZ (2006). Matrix metalloproteinase 2 (MMP2) and MMP9 secreted by erythropoietin-activated endothelial cells promote neural progenitor cell migration. *Journal of Neuroscience*.

[111] Teng H, Zhang ZG, Wang L (2008). Coupling of angiogenesis and neurogenesis in cultured endothelial cells and neural progenitor cells after stroke. *Journal of Cerebral Blood Flow and Metabolism*.

[112] Wang L, Chopp M, Gregg SR (2008). Neural progenitor cells treated with EPO induce angiogenesis through the production of VEGF. *Journal of Cerebral Blood Flow and Metabolism*.

[113] Meng H, Zhang Z, Zhang R (2006). Biphasic effects of exogenous VEGF on VEGF expression of adult neural progenitors. *Neuroscience Letters*.

[114] Faraci FM (1990). Role of nitric oxide in regulation of basilar artery tone in vivo. *American Journal of Physiology*.

[115] Baumbach GL, Sigmund CD, Faraci FM (2004). Structure of cerebral arterioles in mice deficient in expression of the gene for endothelial nitric oxide synthase. *Circulation Research*.

[116] Kitaura H, Uozumi N, Tohmi M (2007). Roles of nitric oxide as a vasodilator in neurovascular coupling of mouse somatosensory cortex. *Neuroscience Research*.

[117] Stefanovic B, Schwindt W, Hoehn M, Silva AC (2007). Functional uncoupling of hemodynamic from neuronal response by inhibition of neuronal nitric oxide synthase. *Journal of Cerebral Blood Flow and Metabolism*.

[118] Genc S, Kuralay F, Genc K (2001). Erythropoietin exerts neuroprotection in 1-methyl-4-phenyl-1,2,3,6-tetrahydropyridine-treated C57/BL mice via increasing nitric oxide production. *Neuroscience Letters*.

[119] Chen ZY, Wang L, Asavaritkrai P, Noguchi CT (2010). Up-regulation of erythropoietin receptor by nitric oxide mediates hypoxia preconditioning. *Journal of Neuroscience Research*.

[120] Tian J, Kim SF, Hester L, Snyder SH (2008). S-nitrosylation/activation of COX-2 mediates NMDA neurotoxicity. *Proceedings of the National Academy of Sciences of the United States of America*.

[121] Kim SF, Huri DA, Snyder SH (2005). Medicine: inducible nitric oxide synthase binds, S-nitrosylates, and activates cyclooxygenase-2. *Science*.

[122] Iadecola C, Zhang F, Casey R, Nagayama M, Elizabeth Ross M (1997). Delayed reduction of ischemic brain injury and neurological deficits in mice lacking the inducible nitric oxide synthase gene. *Journal of Neuroscience*.

[123] Huang Z, Huang PL, Ma J (1996). Enlarged infarcts in endothelial nitric oxide synthase knockout mice are attenuated by nitro-L-arginine. *Journal of Cerebral Blood Flow and Metabolism*.

[124] Dawson VL, Kizushi VM, Huang PL, Snyder SH, Dawson TM (1996). Resistance to neurotoxicity in cortical cultures from neuronal nitric oxide synthase-deficient mice. *Journal of Neuroscience*.

[125] Keswani SC, Bosch-Marcé M, Reed N, Fischer A, Semenza GL, Höke A (2011). Nitric oxide prevents axonal degeneration by inducing HIF-1-dependent expression of erythropoietin. *Proceedings of the National Academy of Sciences of the United States of America*.

[126] Xenocostas A, Cheung WK, Farrell F (2005). The pharmacokinetics of erythropoietin in the cerebrospinal fluid after intravenous administration of recombinant human erythropoietin. *European Journal of Clinical Pharmacology*.

[127] Sakanaka M, Wen TC, Matsuda S (1998). In vivo evidence that erythropoietin protects neurons from ischemic damage. *Proceedings of the National Academy of Sciences of the United States of America*.

[128] Sadamoto Y, Igase K, Sakanaka M (1998). Erythropoietin prevents place navigation disability and cortical infarction in rats with permanent occlusion of the middle cerebral artery. *Biochemical and Biophysical Research Communications*.

[129] Bernaudin M, Nedelec A-S, Divoux D, MacKenzie ET, Petit E, Schumann-Bard P (2002). Normobaric hypoxia induces tolerance to focal permanent cerebral ischemia in association with an increased expression of hypoxia-inducible factor-1 and its target genes, erythropoietin and VEGF, in the adult mouse brain. *Journal of Cerebral Blood Flow and Metabolism*.

[130] Xiong Y, Chopp M, Lee CP (2009). Erythropoietin improves brain mitochondrial function in rats after traumatic brain injury. *Neurological Research*.

[131] Zhang Y, Xiong Y, Mahmood A (2009). Therapeutic effects of erythropoietin on histological and functional outcomes following traumatic brain injury in rats are independent of hematocrit. *Brain Research*.

[132] Taoufik E, Petit E, Divoux D (2008). TNF receptor I sensitizes neurons to erythropoietin-and VEGF-mediated neuroprotection after ischemic and excitotoxic injury. *Proceedings of the National Academy of Sciences of the United States of America*.

[133] Yamada M, Burke C, Colditz P, Johnson DW, Gobe GC (2011). Erythropoietin protects against apoptosis and increases expression of non-neuronal cell markers in the hypoxia-injured developing brain. *Journal of Pathology*.

[134] Gunnarson E, Song Y, Kowalewski JM (2009). Erythropoietin modulation of astrocyte water permeability as a component of neuroprotection. *Proceedings of the National Academy of Sciences of the United States of America*.

[135] Krügel K, Wurm A, Linnertz R (2010). Erythropoietin inhibits osmotic swelling of retinal glial cells by Janus kinase- and extracellular signal-regulated kinases1/2-mediated release of vascular endothelial growth factor. *Neuroscience*.

[136] Prass K, Scharff A, Ruscher K (2003). Hypoxia-induced stroke tolerance in the mouse is mediated by erythropoietin. *Stroke*.

[137] Malhotra S, Savitz SI, Ocava L, Rosenbaum DM (2006). Ischemic preconditioning is mediated by erythropoietin through PI-3 kinase signaling in an animal model of transient ischemic attack. *Journal of Neuroscience Research*.

[138] Theus MH, Wei L, Cui L (2008). In vitro hypoxic preconditioning of embryonic stem cells as a strategy of promoting cell survival and functional benefits after transplantation into the ischemic rat brain. *Experimental Neurology*.

[139] Zaman K, Ryu H, Hall D (1999). Protection from oxidative stress-induced apoptosis in cortical neuronal cultures by iron chelators is associated with enhanced DNA binding of hypoxia-inducible factor-1 and ATF-1/CREB and increased expression of glycolytic enzymes, p21(waf1/cip1), and erythropoietin. *Journal of Neuroscience*.

[140] Siddiq A, Ayoub IA, Chavez JC (2005). Hypoxia-inducible factor prolyl 4-hydroxylase inhibition: a target for neuroprotection in the central nervous system. *Journal of Biological Chemistry*.

[141] Liu J, Narasimhan P, Yu F, Chan PH (2005). Neuroprotection by hypoxic preconditioning involves oxidative stress-mediated expression of hypoxia-inducible factor and erythropoietin. *Stroke*.

[142] Milosevic J, Maisel M, Wegner F (2007). Lack of hypoxia-inducible factor-1*α* impairs midbrain neural precursor cells involving vascular endothelial growth factor signaling. *Journal of Neuroscience*.

[143] Leconte C, Tixier E, Freret T (2009). Delayed hypoxic postconditioning protects against cerebral ischemia in the mouse. *Stroke*.

[144] Aydin A, Genç K, Akhisaroglu M, Yorukoglu K, Gokmen N, Gonullu E (2003). Erythropoietin exerts neuroprotective effect in neonatal rat model of hypoxic-ischemic brain injury. *Brain and Development*.

[145] Matsushita H, Johnston MV, Lange MS, Wilson MA (2003). Protective effect of erythropoietin in neonatal hypoxic ischemia in mice. *NeuroReport*.

[146] Wen TC, Rogido M, Peng H, Genetta T, Moore J, Sola A (2006). Gender differences in long-term beneficial effects of erythropoietin given after neonatal stroke in postnatal day-7 rats. *Neuroscience*.

[147] Kumral A, Uysal N, Tugyan K (2004). Erythropoietin improves long-term spatial memory deficits and brain injury following neonatal hypoxia-ischemia in rats. *Behavioural Brain Research*.

[148] Spandou E, Soubasi V, Papoutsopoulou S (2004). Erythropoietin prevents hypoxia/ischemia-induced DNA fragmentation in an experimental model of perinatal asphyxia. *Neuroscience Letters*.

[149] Sun Y, Zhou C, Polk P, Nanda A, Zhang JH (2004). Mechanisms of Erythropoietin-induced Brain Protection in Neonatal Hypoxia-Ischemia Rat Model. *Journal of Cerebral Blood Flow and Metabolism*.

[150] Demers EJ, McPherson RJ, Juul SE (2005). Erythropoietin protects dopaminergic neurons and improves neurobehavioral outcomes in juvenile rats after neonatal hypoxia-ischemia. *Pediatric Research*.

[151] Gonzalez FF, McQuillen P, Mu D (2007). Erythropoietin enhances long-term neuroprotection and neurogenesis in neonatal stroke. *Developmental Neuroscience*.

[152] Spandou E, Papadopoulou Z, Soubasi V (2005). Erythropoietin prevents long-term sensorimotor deficits and brain injury following neonatal hypoxia-ischemia in rats. *Brain Research*.

[153] McClure MM, Threlkeld SW, Fitch RH (2007). Auditory processing and learning/memory following erythropoietin administration in neonatally hypoxic-ischemic injured rats. *Brain Research*.

[154] Spandou E, Papoutsopoulou S, Soubasi V (2004). Hypoxia-ischemia affects erythropoietin and erythropoietin receptor expression pattern in the neonatal rat brain. *Brain Research*.

[155] Wen TC, Rogido M, Genetta T, Sola A (2004). Permanent focal cerebral ischemia activates erythropoietin receptor in the neonatal rat brain. *Neuroscience Letters*.

[156] Yiş U, Kurul SH, Kumral A (2008). Effect of erythropoietin on oxygen-induced brain injury in the newborn rat. *Neuroscience Letters*.

[157] Kaindl AM, Sifringer M, Koppelstaetter A (2008). Erythropoietin protects the developing brain from hyperoxia-induced cell death and proteome changes. *Annals of Neurology*.

[158] Juul SE, Beyer RP, Bammler TK, Mcpherson RJ, Wilkerson J, Farin FM (2009). Microarray analysis of high-dose recombinant erythropoietin treatment of unilateral brain injury in neonatal mouse hippocampus. *Pediatric Research*.

[159] Juul SE, McPherson RJ, Bammler TK, Wilkerson J, Beyer RP, Farin FM (2008). Recombinant erythropoietin is neuroprotective in a novel mouse oxidative injury model. *Developmental Neuroscience*.

[160] Kumral A, Baskin H, Yesilirmak DC (2007). Erythropoietin attenuates lipopolysaccharide-induced white matter injury in the neonatal rat brain. *Neonatology*.

[161] Shen Y, Yu HM, Yuan TM, Gu WZ, Wu YD (2009). Erythropoietin attenuates white matter damage, proinflammatory cytokine and chemokine induction in developing rat brain after intra-uterine infection. *Neuropathology*.

[162] Kellert BA, McPherson RJ, Juul SE (2007). A comparison of high-dose recombinant erythropoietin treatment regimens in brain-injured neonatal rats. *Pediatric Research*.

[163] Mazur M, Miller RH, Robinson S (2010). Postnatal erythropoietin treatment mitigates neural cell loss after systemic prenatal hypoxic-ischemic injury: laboratory investigation. *Journal of Neurosurgery: Pediatrics*.

[164] Xiong Y, Lu D, Qu C (2008). Effects of erythropoietin on reducing brain damage and improving functional outcome after traumatic brain injury in mice. *Journal of Neurosurgery*.

[165] Iwai M, Stetler RA, Xing J (2010). Enhanced oligodendrogenesis and recovery of neurological function by erythropoietin after neonatal hypoxic/ischemic brain injury. *Stroke*.

[166] Juul SE, McPherson RJ, Bauer LA, Ledbetter KJ, Gleason CA, Mayock DE (2008). A phase I/II trial of high-dose erythropoietin in extremely low birth weight infants: pharmacokinetics and safety. *Pediatrics*.

[167] Fauchère JC, Dame C, Vonthein R (2008). An approach to using recombinant erythropoietin for neuroprotection in very preterm infants. *Pediatrics*.

[168] Villa P, Bigini P, Mennini T (2003). Erythropoietin selectively attenuates cytokine production and inflammation in cerebral ischemia by targeting neuronal apoptosis. *Journal of Experimental Medicine*.

[169] Dirnagl U, Iadecola C, Moskowitz MA (1999). Pathobiology of ischaemic stroke: an integrated view. *Trends in Neurosciences*.

[170] Witko-Sarsat V, Rieu P, Descamps-Latscha B, Lesavre P, Halbwachs-Mecarelli L (2000). Neutrophils: molecules, functions and pathophysiological aspects. *Laboratory Investigation*.

[171] Zhang J, Li Y, Cui Y (2005). Erythropoietin treatment improves neurological functional recovery in EAE mice. *Brain Research*.

[172] Savino C, Pedotti R, Baggi F (2006). Delayed administration of erythropoietin and its non-erythropoietic derivatives ameliorates chronic murine autoimmune encephalomyelitis. *Journal of Neuroimmunology*.

[173] Agnello D, Bigini P, Villa P (2002). Erythropoietin exerts an anti-inflammatory effect on the CNS in a model of experimental autoimmune encephalomyelitis. *Brain Research*.

[174] Yuan R, Maeda Y, Li W, Lu W, Cook S, Dowling P (2008). Erythropoietin: a potent inducer of peripheral immuno/inflammatory modulation in autoimmune EAE. *PLoS One*.

[175] Sättler MB, Merkler D, Maier K (2004). Neuroprotective effects and intracellular signaling pathways of erythropoietin in a rat model of multiple sclerosis. *Cell Death and Differentiation*.

[176] Genc K, Genc S, Baskin H, Semin I (2006). Erythropoietin decreases cytotoxicity and nitric oxide formation induced by inflammatory stimuli in rat oligodendrocytes. *Physiological Research*.

[177] Patel S, Rowe MJ, Winters SA, Ohls RK (2008). Elevated erythropoietin mRNA and protein concentrations in the developing human eye. *Pediatric Research*.

[178] Watanabe D, Suzuma K, Matsui S (2005). Erythropoietin as a retinal angiogenic factor in proliferative diabetic retinopathy. *New England Journal of Medicine*.

[179] Tong Z, Yang Z, Patel S (2008). Promoter polymorphism of the erythropoietin gene in severe diabetic eye and kidney complications. *Proceedings of the National Academy of Sciences of the United States of America*.

[180] Scheerer N, Dünker N, Imagawa S, Yamamoto M, Suzuki N, Fandrey J (2010). The anemia of the newborn induces erythropoietin expression in the developing mouse retina. *American Journal of Physiology*.

[181] Grimm C, Wenzel A, Stanescu D (2004). Constitutive overexpression of human erythropoietin protects the mouse retina against induced but not inherited retinal degeneration. *Journal of Neuroscience*.

[182] Chen J, Connor KM, Aderman CM, Smith LEH (2008). Erythropoietin deficiency decreases vascular stability in mice. *Journal of Clinical Investigation*.

[183] Ehrenreich H, Hasselblatt M, Dembowski C (2002). Erythropoietin therapy for acute stroke is both safe and beneficial. *Molecular Medicine*.

[184] Ehrenreich H, Weissenborn K, Prange H (2009). Recombinant human erythropoietin in the treatment of acute ischemic stroke. *Stroke*.

[185] Jia L, Chopp M, Zhang L, Lu M, Zhang Z (2010). Erythropoietin in combination of tissue plasminogen activator exacerbates brain hemorrhage when treatment is initiated 6 hours after stroke. *Stroke*.

[186] Zechariah A, Elali A, Hermann DM (2010). Combination of tissue-plasminogen activator with erythropoietin induces blood-brain barrier permeability, extracellular matrix disaggregation, and DNA fragmentation after focal cerebral ischemia in mice. *Stroke*.

[187] Meng Y, Xiong Y, Mahmood A, Zhang Y, Changsheng Q, Chopp M (2011). Dose-dependent neurorestorative effects of delayed treatment of traumatic brain injury with recombinant human erythropoietin in rats: laboratory investigation. *Journal of Neurosurgery*.

[188] Pfeffer MA, Burdmann EA, Chen CY (2009). A trial of darbepoetin alfa in type 2 diabetes and chronic kidney disease. *New England Journal of Medicine*.

[189] Singh AK, Szczech L, Tang KL (2006). Correction of anemia with epoetin alfa in chronic kidney disease. *New England Journal of Medicine*.

[190] Besarab A, Bolton WK, Browne JK (1998). The effects of normal as compared with low hematocrit values in patients with cardiac disease who are receiving hemodialysis and epoetin. *New England Journal of Medicine*.

[191] Solomon SD, Uno H, Lewis EF (2010). Erythropoietic response and outcomes in kidney disease and type 2 diabetes. *New England Journal of Medicine*.

[192] Leyland-Jones B (2003). Breast cancer trial with erythropoietin terminated unexpectedly. *Lancet Oncology*.

[193] Henke M, Laszig R, Rübe C (2003). Erythropoietin to treat head and neck cancer patients with anaemia undergoing radiotherapy: randomised, double-blind, placebo-controlled trial. *Lancet*.

[194] Bennett CL, Silver SM, Djulbegovic B (2008). Venous thromboembolism and mortality associated with recombinant erythropoietin and darbepoetin administration for the treatment of cancer-associated anemia. *Journal of the American Medical Association*.

[195] Erbayraktar S, Grasso G, Sfacteria A (2003). Asialoerythropoietin is a nonerythropoietic cytokine with broad neuroprotective activity in vivo. *Proceedings of the National Academy of Sciences of the United States of America*.

[196] Yip H-K, Tsai T-H, Lin H-S (2011). Effect of erythropoietin on level of circulating endothelial progenitor cells and outcome in patients after acute ischemic stroke. *Critical Care*.

[197] Cramer SC, Fitzpatrick C, Warren M (2010). The beta-hCG+erythropoietin in acute stroke (BETAS) study: a 3-center, single-dose, open-label, noncontrolled, phase IIa safety trial. *Stroke*.

[198] Wüstenberg T, Begemann M, Bartels C (2011). Recombinant human erythropoietin delays loss of gray matter in chronic schizophrenia. *Molecular Psychiatry*.

[199] Leis M, Gliezzi P, Grasso G (2004). Derivatives of erythropoietin that are tissue protective but not erythropoietic. *Science*.

[200] Teng R, Gavrilova O, Suzuki N (2011). Disrupted erythropoeitin signaling promotes obesity and alters hypothalamus proopiomelanocortin production. *Nature Communications*.

[201] Foskett A, Alnaeeli M, Wang L, Teng R, Noguchi CT (2011). The effects of erythropoietin dose titration during high-fat diet-induced obesity. *Journal of Biomedicine and Biotechnology*.

[202] Katz O, Stuible M, Golishevski N (2010). Erythropoietin treatment leads to reduced blood glucose levels and body mass: insights from murine models. *Journal of Endocrinology*.

[203] Hojman P, Brolin C, Gissel H (2009). Erythropoietin over-expression protects against diet-induced obesity in mice through increased fat oxidation in muscles. *PLoS One*.

[204] Choi D, Schroer SA, Lu SY (2010). Erythropoietin protects against diabetes through direct effects on pancreatic *β* cells. *Journal of Experimental Medicine*.

